# Tetraphenylethylene-embedded pillar[5]arene-based orthogonal self-assembly for efficient photocatalysis in water

**DOI:** 10.3762/bjoc.18.45

**Published:** 2022-04-13

**Authors:** Zhihang Bai, Krishnasamy Velmurugan, Xueqi Tian, Minzan Zuo, Kaiya Wang, Xiao-Yu Hu

**Affiliations:** 1 College of Materials Science and Technology, Nanjing University of Aeronautics and Astronautics, Nanjing 211106, P. R. China

**Keywords:** aggregation-induced emission, Förster resonance energy transfer, host–guest interaction, photocatalysis, supramolecular self-assembly

## Abstract

Herein, we have designed and fabricated a simple and efficient supramolecular self-assembled nanosystem based on host–guest interactions between water-soluble tetraphenylethylene-embedded pillar[5]arene (***m*-TPEWP5**) and ammonium benzoyl-ʟ-alaninate (**G**) in an aqueous medium. The obtained assembly of ***m*-TPEWP5** and **G** showed aggregation-induced emission (AIE) via the blocking of intramolecular phenyl-ring rotations and functioned as an ideal donor. After the loading of eosin Y (**EsY**) as acceptor on the surface of the assembly of ***m*-TPEWP5** and **G**, the worm-like nanostructures changed into nanorods, which facilitates a Förster resonance energy transfer (FRET) from the ***m*-TPEWP5** and **G** assembled donor to the **EsY** acceptor present in the nanorod assembly. The system comprising ***m*-TPEWP5**, **G** and **EsY** displayed moderate FRET efficiency (31%) at a 2:1 molar ratio of donor-to-acceptor. Moreover, the obtained supramolecular nanorod assembly could act as a nanoreactor mimicking natural photosynthesis and exhibited a high catalytic efficiency for the photocatalytic dehalogenation reaction of various bromoketone derivatives with good yields in short reaction time in water.

## Introduction

Photosynthesis is one of the most significant processes in nature, which balances the energy level in living systems [1–3]. In particular, green plants absorb photons of light and convert them into another form of energy through photosynthesis similar to solar power factories, containing many manufacturing units called chloroplasts. Briefly, the antenna molecules capture the light energy by using protein–pigment complexes and transfer it to the specialized reaction centers via the FRET process, where the excited state energy is transferred into useable chemical energy [4–6]. Mainly, both antenna molecules and proteins on the thylakoid membrane are combined to form a light-harvesting system through noncovalent interactions. Inspired by photosynthesis, extensive research has been devoted to construct energy transfer systems for the better utilization of solar energy [7]. In general, an effective supramolecular donor–acceptor system was employed to construct a photocatalytic system using FRET [6,8]. To fabricate a successful FRET system, the following key points need to be considered, i) the acceptor absorption spectrum should have good overlapping with the donor emission spectrum; ii) the distance between the donor and an acceptor should be within 10 nm; iii) dipoles of the donor and acceptor molecules must be adopted constructively vicinity to each other [9]. These fundamental criteria provide a wonderful approach for the construction of supramolecular photocatalytic systems by self-assembly strategies [10–11].

Recently, FRET-based supramolecular self-assembled systems [12–13] as nanoreactors for various photocatalytic reactions have received significant attention from the supramolecular community because of their robust molecular design and tunable self-assembly, such as vesicles [14–16], micelles [17–19], nanocrystals [20], coordination-driven assemblies [9,21–22], host–guest interactions [15,23–25], etc. In the above systems, catalysts are encapsulated by supramolecular assemblies and thus provide a suitable environment to improve the efficiency of chemical reactions in water [26]. Until now, various macrocyclic host-assisted supramolecular donor–acceptor systems have been developed based on the FRET process and further utilized for different photochemical reactions [27–28]. For instance, Yi et al. [29] developed a supramolecular assembly with a two-step FRET process by the utilization of a metallacycle-tetraphenylethylene (TPE) donor and eosin Y (EsY) and sulforhodamine (SR101) as first and second acceptors, respectively. The resulting supramolecular energy transfer system was applied to the alkylation of C–H bonds via a photochemical catalytic reaction in aqueous medium. In addition, our group [30] reported the construction of a supramolecular photocatalytic system with a two-step FRET process through the supramolecular assembly of water-soluble pillar[5]arene and TPE derivatives as donor and EsY and Nile Red (NiR) as acceptors. The obtained vesicles could be utilized as a nanoreactor for photocatalyzed dehalogenation reactions in water. However, the above reported supramolecular nanosystem requires a long time to produce the dehalogenated product with high yield. Therefore, the development of a potential nanoreactor for dehalogenation reaction with high yields within shorter reaction time is vastly essential and of industrial importance.

**Scheme 1:**
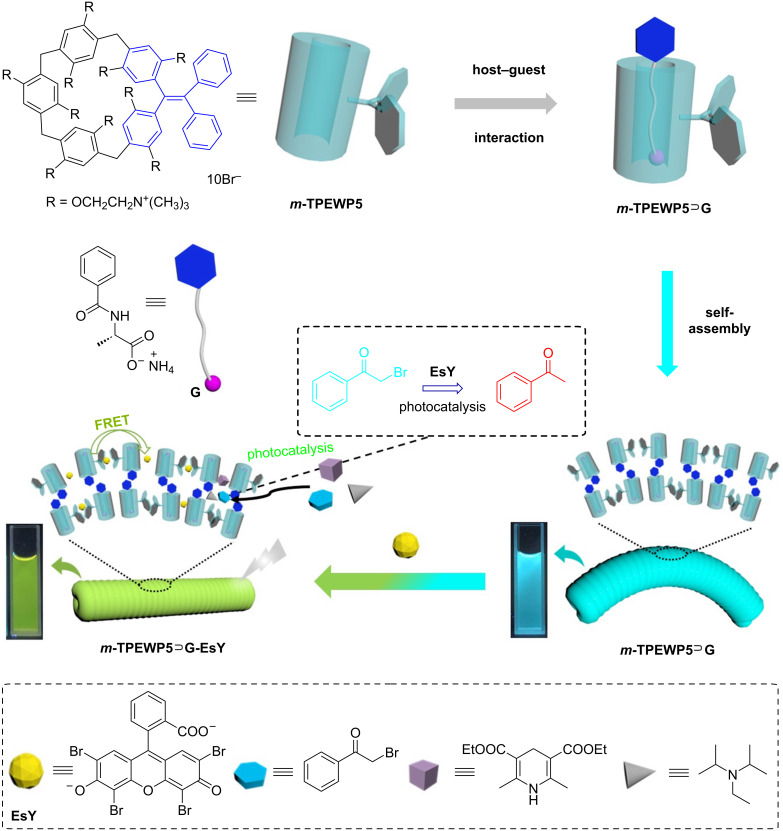
Graphical representation of the fabrication of supramolecular ***m*-TPEWP5**
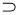
**G-EsY** self-assembled photocatalytic system.

Herein, we have fabricated a supramolecular AIE-emissive photocatalytic system (***m*-TPEWP5**
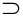
**G–EsY**) based on the host–guest interactions between *meso*-TPE embedded water-soluble pillar[5]arene (***m*-TPEWP5**) as host and the guest ammonium benzoyl-ʟ-alaninate (**G**) forming the ***m*-TPEWP5**
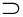
**G** complex onto which **EsY** was loaded to achieve moderate FRET efficiency in water (Scheme 1). When the guest **G** was added to the host ***m*-TPEWP5** a stable host–guest complex formed, which strongly inhibited the intramolecular phenyl-ring rotations thus enhancing the AIE property. The resulting ***m*-TPEWP5**
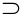
**G** self-assembled worm-like nanosystem acts as an ideal donor and loading **EsY** as acceptor on the surface of the worm-like nanostructure, leads to the generation of a nanorod assembly via electrostatic interactions. The final AIE-emissive ***m*-TPEWP5**
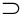
**G**-**EsY** self-assembled FRET system could be employed to promote the photocatalyzed dehalogenation of various haloketone derivatives with excellent yields in water.

## Results and Discussion

**Figure 1:**
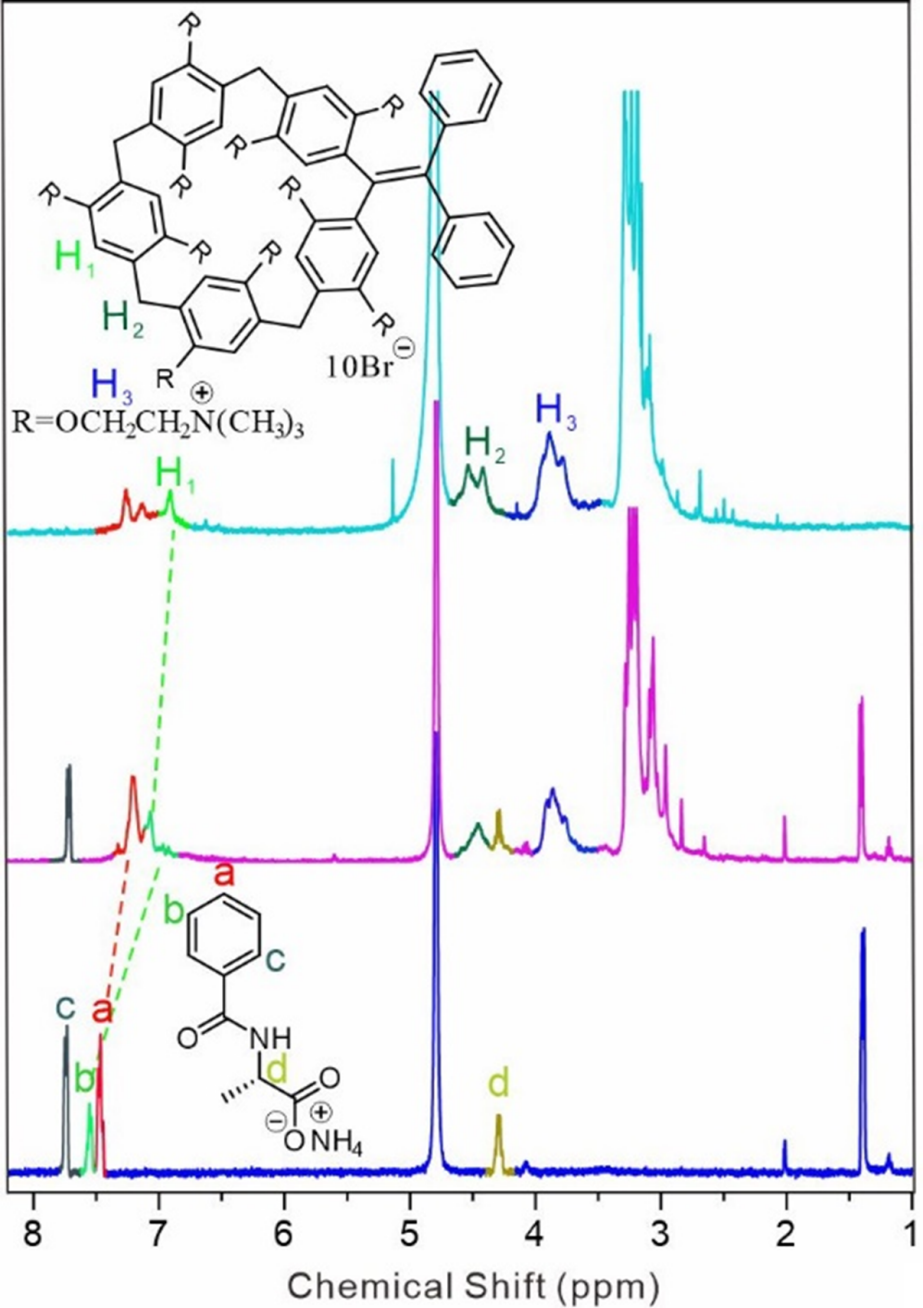
^1^H NMR (400 MHz, D_2_O, 298 K) spectra of ***m*-TPEWP5** (1.0 mM), ***m*-TPEWP5** (1.0 mM) + **G** (1.0 mM), and **G** (1.0 mM).

The ***m*-TPEWP5** host and ammonium benzoyl-ʟ-alaninate (**G**) were synthesized according to our previous work [31–32] and their detailed synthetic routes and characterization data are provided in Supporting Information File 1 (Figure S1). Since ***m*-TPEWP5** and **G** have good solubility in water, therefore, the ***m*-TPEWP5**
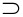
**G** and ***m*-TPEWP5**
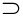
**G-EsY** supramolecular assemblies could be potentially fabricated in an aqueous solution. Before studying the FRET process, we firstly investigated the host–guest interactions between ***m*-TPEWP5** and **G** in D_2_O. Upon the addition of **G** (1 equiv) to ***m*-TPEWP5**, the resonance peaks of **G** containing H_a_ and H_b_ protons shifted to the upfield region in the NMR scale (Figure 1). Meanwhile, the ***m*-TPEWP5** aromatic H_1_ proton signal shifted to the downfield region, displaying that the guest molecule has a good binding affinity with the ***m*-TPEWP5** host to form a stable host–guest complex. In addition, 2D NOESY NMR (Figure S2 in Supporting Information File 1) was carried out to further confirm the interaction between ***m*-TPEWP5** and **G** (1 equiv of each) in D_2_O. A strong cross-correlation peak was perceived between the ***m*-TPEWP5** aromatic protons and the H_c_ proton of **G**. The above results evidenced that the alanine pendant of the guest unit stayed in the ***m*-TPEWP5** cavity.

**Figure 2:**
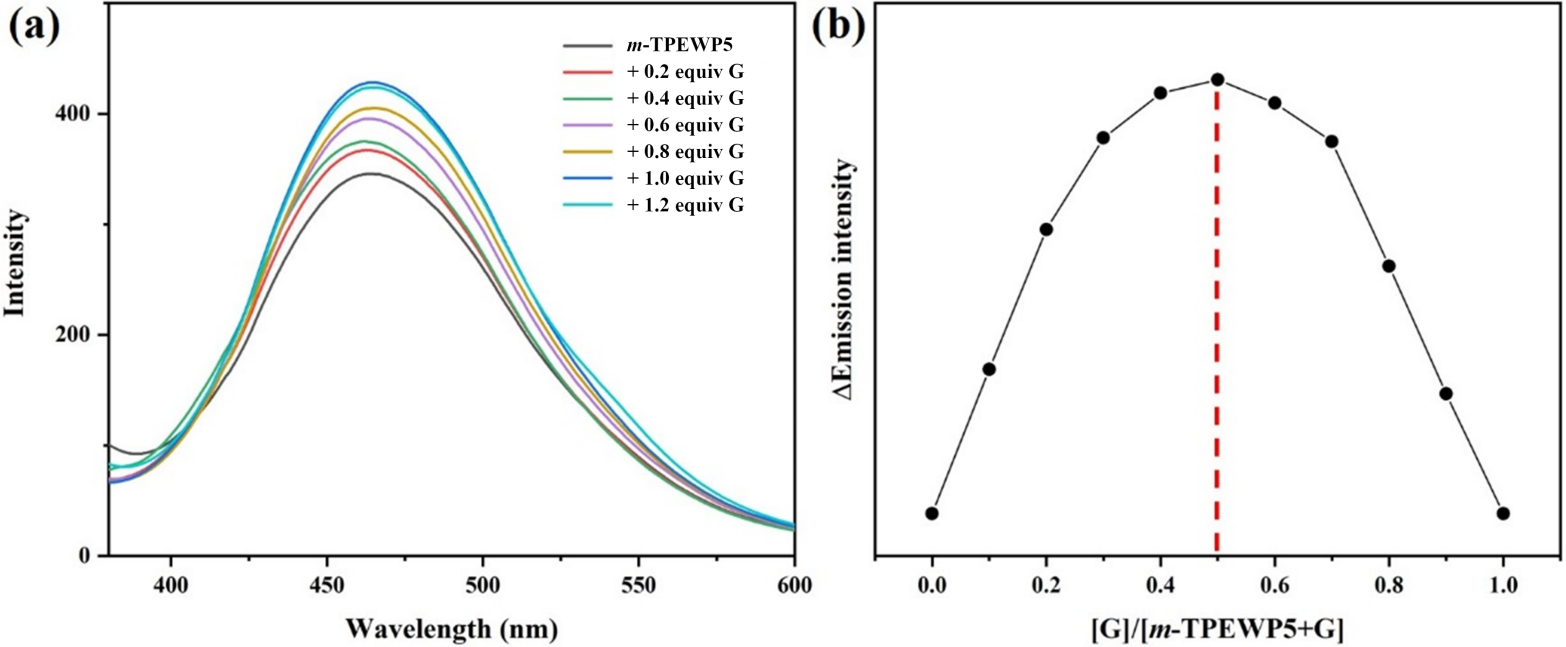
(a) Fluorescence spectra of ***m*-TPEWP5** (1 × 10^−5^ M) with different concentrations of **G** (0 to 1.2 equiv); (b) Job’s plot of ***m*-TPEWP5**
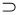
**G** showing a 1:1 binding stoichiometry between ***m*-TPEWP5** and **G** by plotting the emission intensity differences at 470 nm against the mole fraction of **G** in an aqueous solution (λ_ex_ = 320 nm).

Besides, in order to confirm the host–guest interactions between ***m*-TPEWP5** and **G**, fluorescence titration studies were carried out in aqueous solution. As shown in Figure 2a, the free host ***m*-TPEWP5** showed a maximum emission at 465 nm. Upon gradually increasing the concentration of **G** (0 to 1 equiv) into ***m*-TPEWP5**, the fluorescence intensities were significantly increased with respect to **G** concentrations and no considerable changes were observed when further increasing the **G** concentration (1.2 equiv). The above results corroborated that the free rotation of ***m*-TPEWP5** rings was arrested during the complexation with **G**, thus further leads to the enhanced AIE effect [33–34].

To examine the binding stoichiometric ratio of the host–guest complex, Job’s plot [35] method was employed by using fluorescence titration experiments. As shown in Figure S3 (see Supporting Information File 1), the maximum mole fraction was observed at 0.5 (Figure 2b), which corresponds to a 1:1 binding stoichiometric ratio between **G** and ***m*-TPEWP5** in the aqueous solution. Furthermore, the association constant (*K*_a_) [36] was calculated to be 8.62 × 10^4^ M^−1^ based on the UV–vis titration experiment (Figure S4, Supporting Information File 1). This result further confirmed that the binding interaction between ***m*-TPEWP5** and **G** is strong enough to form a stable complex in an aqueous solution.

**Figure 3:**
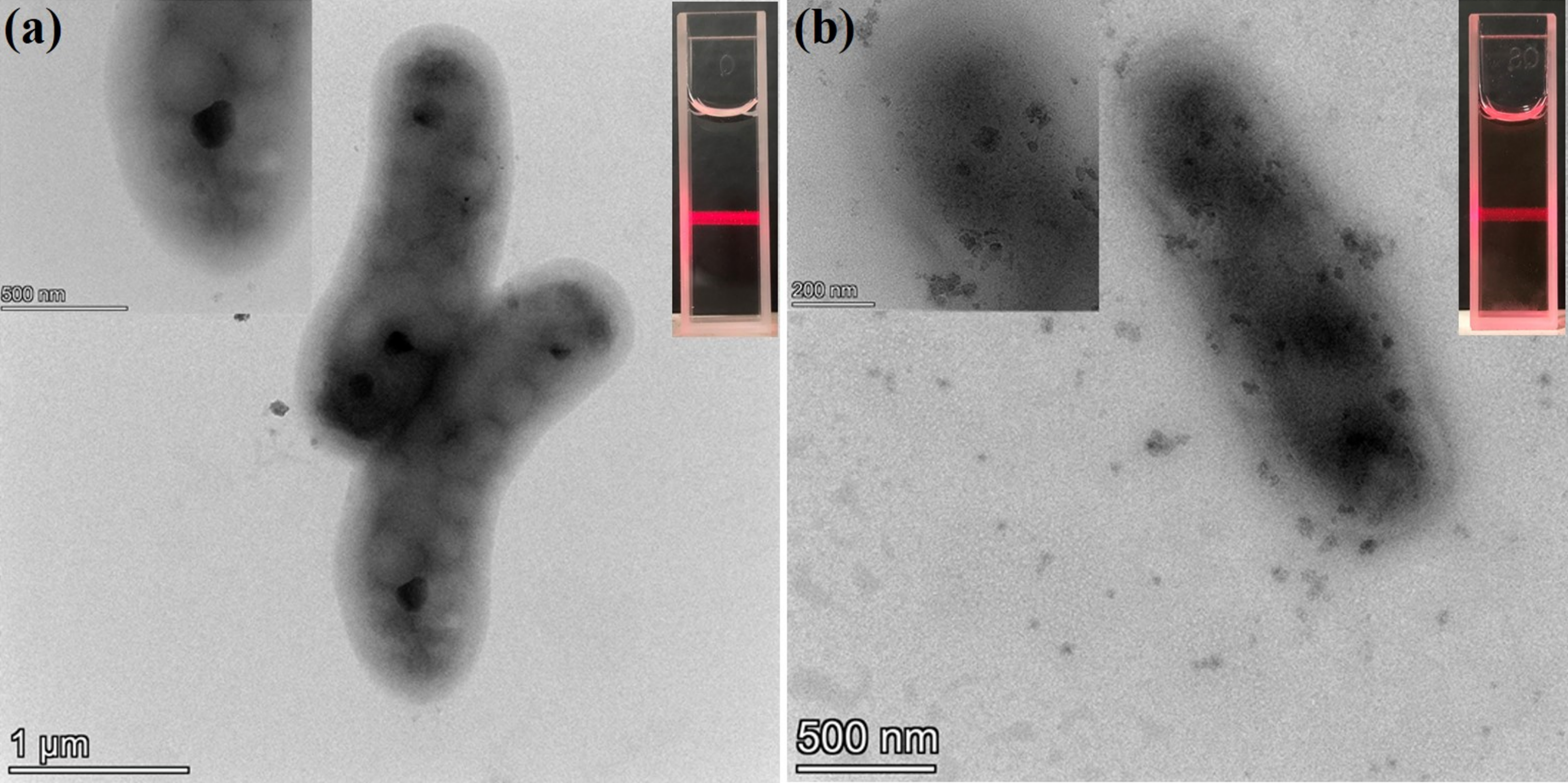
TEM images of (a) ***m*-TPEWP5**
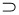
**G**; (b) ***m*-TPEWP5**
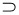
**G-EsY**. [***m*-TPEWP5**] = 1 × 10^−4^ M, [**G**] = 1 × 10^−4^ M, [**EsY**] = 1 × 10^−4^ M.

The morphology of the supramolecular ***m*-TPEWP5**
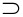
**G** and ***m*-TPEWP5**
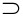
**G-EsY** systems was monitored by using transmission electron microscopy (TEM). As shown in Figure 3, ***m*-TPEWP5**
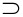
**G** self-assembled to form a worm-like nanostructure (diameter = 748 nm). After the loading of **EsY** into ***m*-TPEWP5**
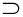
**G**, the worm-like structure changed into a nanorod (diameter = 652 nm) assembly via electrostatic interactions. In comparison, the diameter and length of the ***m*-TPEWP5**
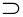
**G** assembly were slightly higher than that of ***m*-TPEWP5**
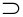
**G-EsY**, which revealed that host–guest complex aggregated to form a stable structural assembly, then a dye-loaded composite system [32].

**Figure 4:**
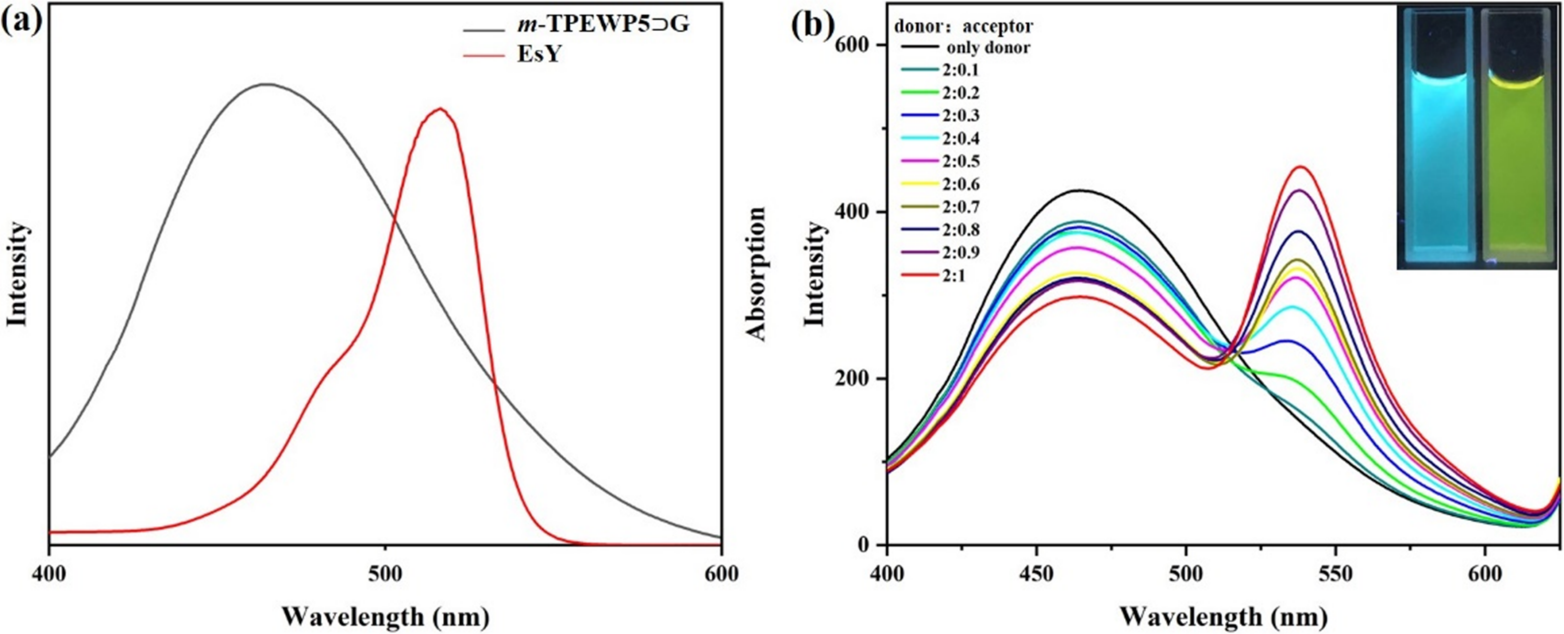
(a) Normalized absorption and emission spectra of the **EsY** acceptor and the ***m*-TPEWP5**
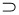
**G** donor assembly; (b) Fluorescence spectra of the ***m*-TPEWP5**
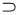
**G** assembly with steadily increasing the equivalents of **EsY** in an aqueous medium (λ_ex._ = 320 nm). Inset: Photograph of ***m*-TPEWP5**
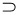
**G** before and after the addition of **EsY** under UV light.

The energy transfer efficiency of the supramolecular ***m*-TPEWP5**
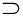
**G-EsY** self-assembled composite system was examined by using ***m*-TPEWP5**
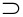
**G** and **EsY** as an ideal donor and acceptor, respectively. Initially, we compared the overlapping efficiencies of both donor and acceptor systems by using UV–vis and fluorescence spectroscopy. The absorption band of the **EsY** acceptor shows good overlapping with the emission band of ***m*-TPEWP5**
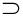
**G** donor (Figure 4a). We therefore speculated, that there may be an efficient FRET process between the donor and acceptor containing nanorod assembly. As shown in Figure 4b, upon exciting at the donor wavelength (λ_ex_ = 320 nm), the fluorescence intensity of the ***m*-TPEWP5**
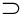
**G** donor gradually decreased, whereas the **EsY** acceptor emission peak at 540 nm gradually increased thus indicating an efficient energy transfer is taking place. This was further supported by the observation, that upon loading of **EsY** into the ***m*-TPEWP5**
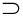
**G** assembly, the color of the donor solution changed from sky blue to greenish-yellow under UV light. At a 2:1 donor/acceptor molar ratio, a maximum FRET efficiency of 31% was achieved [30]. This result suggested that ***m*-TPEWP5**
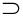
**G-EsY** self-assembled nanosystem could be used as a nanoreactor for organic photocatalytic reactions in an aqueous medium. The nature of the interaction between ***m*-TPEWP5**
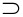
**G** and **EsY** was evaluated by ^1^H NMR titration studies in D_2_O (Figure S5 in Supporting Information File 1). When 1 equiv of **EsY** was added into the ***m*-TPEWP5**
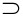
**G** solution, the resonance signals of **EsY** were shifted to upfield regions, which was caused by steric effects and electrostatic interactions between the quaternary ammonium groups containing ***m*-TPEWP5** and the negatively charged **EsY**. These results evidenced that the **EsY** molecule was adsorbed on the surface of ***m*-TPEWP5** via electrostatic interactions.

To mimic natural photosynthesis, the harvested energy of the ***m*-TPEWP5**
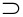
**G-EsY** nanoreactor system could potentially be applied to a photocatalytic dehalogenation reaction. Normally, most chromophoric dye molecules can be utilized in photoredox reactions under the irradiation of light with suitable wavelength [6]. However, in the case of the ***m*-TPEWP5**
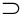
**G-EsY** nanosystem, which contains conjugated molecules and displays harvesting antenna effects from ultraviolet to visible light wavelengths, solar light might be successfully employed to catalyze these reactions. Here, we used white light (20 W) as a solar light simulator for the photocatalytic dehalogenation reactions. Upon light irradiation, the absorbed light energy could be transferred from the ***m*-TPEWP5**
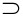
**G** donor to the **EsY** acceptor through the FRET process, whilst the ***m*-TPEWP5**
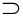
**G-EsY** nanorod assembly could act as a nanoreactor providing a suitable environment for the photochemical catalytic reaction in aqueous solution under visible light irradiation.

In the presence of 0.5 mol % ***m*-TPEWP5**
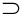
**G-EY** in aqueous solution, 2-bromo-1-phenylethanone (**1a**) gave acetophenone (**2a**) as product in good yield (97%) under white light irradiation for 2 hours (Table 1 and Supporting Information File 1, Figure S6). In comparison, we added an internal standard (1,3,5-trimethoxybenzene) to the final crude reaction mixture and calculated the NMR yield of the product. From the above method, the yield of the product **2a** (97%) is almost identical with our previous results (i.e., assumption of complete conversion of the starting material) as shown in Figure S7 (Supporting Information File 1). Therefore, these results corroborated that there were almost no other byproducts in the system. For further confirmation, we have included the ^13^C NMR spectrum of the crude product **2a** in Supporting Information File 1 (Figure S8).

**Table 1:** 2-Bromo-1-phenylethanone dehalogenation reaction under various reaction conditions.^a^

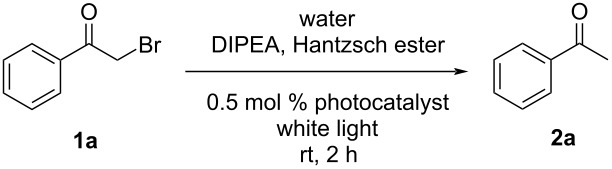			

Entry	Photocatalyst^a^	Light irradiation	Yield^b^ [%]

1	none	yes	28
2	**EsY**	yes	38
3	***m*** **-TPEWP5** 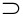 **G**	yes	40
4	***m*** **-TPEWP5** 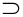 **G-EsY**	yes	97
5^c^	***m*** **-TPEWP5** 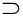 **G-EsY**	no	no reaction

^a^Reaction conditions: Bromoacetophenone (20 mg, 0.1 mmol), Hantzsch ester (28 mg, 0.11 mmol), *N*,*N*-diisopropylethylamine (DIPEA, 35 μL, 0.2 mmol), ***m*-TPEWP5**
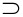
**G-EsY** in water (2.5 mL), 20 W white light, rt, N_2_, 2 h. ^b^Product yield obtained from ^1^H NMR spectra; ^c^Under dark conditions (no light irradiation).

As a control experiment, the reaction was carried out in the absence of catalyst ***m*-TPEWP5**
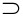
**G** with **EsY** alone (Figure S14, Supporting Information File 1), respectively, and the obtained product yield was very low under light irradiation. Notably, under dark conditions (no light irradiation), there was no product observed in the resulting solution. The above result evidenced that a light source is indispensable for the catalytic dehalogenation reaction in an aqueous environment. Overall, the ***m*-TPEWP5**
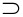
**G-EsY** system showed high catalytic efficiency within a short time of light irradiation in aqueous solution, which was due to the fact that the loaded **EsY** dye molecules on the surface of the **TPEWP5**
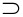
**G** nanorod assembly significantly decreased photobleaching during light irradiation. In addition, the AIE donor molecules allowed an ordered arrangement of the loaded negatively charged dye acceptor on the positively charged surface, which might avoid aggregation-caused quenching effects and produced better catalytic efficiency. These results revealed that the ***m*-TPEWP5**
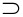
**G-EsY** nanoreactor can act as an effective photocatalytic system to harvest and transform solar energy into chemical energy in aqueous solution.

**Scheme 2:**
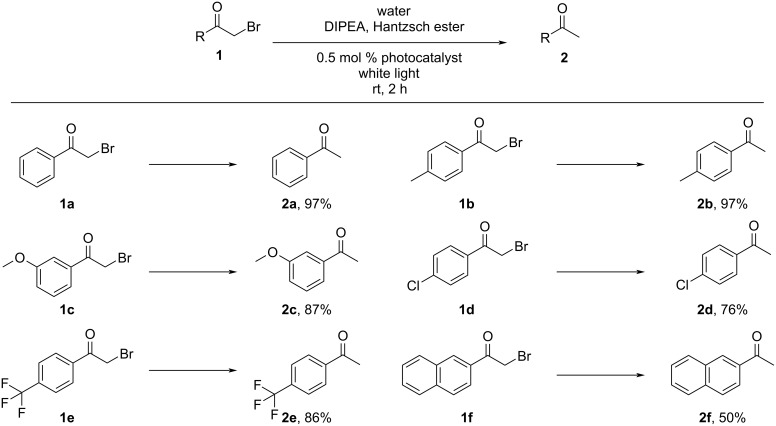
Products from 2-bromo-1-phenylethanone dehalogenation reactions in the presence of ***m*-TPEWP5**
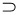
**G-EsY** nanoassembly under white light irradiation. Reaction conditions: Bromoacetophenone (0.1 mmol), Hantzsch ester (28 mg, 0.11 mmol), *N*,*N*-diisopropylethylamine (DIPEA, 35 μL, 0.2 mmol), ***m*-TPEWP5**
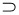
**G-EsY** in water (0.5 mol %, 2.5 mL), 20 W white light, rt, N_2_, 2 h. Product yields were obtained from ^1^H NMR spectra.

Similarly, we carried out the dehalogenation reactions by using various α-bromoacetophenone derivatives as substrates. As shown in Scheme 2, different substrates **1** containing electron-donating (**1b**,**c**) and electron-withdrawing substituents (**1d**,**e**) and 2-bromo-2-acetonaphthone (**1f**) were examined in the reaction. Most substrates afforded the corresponding products in good yields (the ^1^H NMR spectra of the reaction mixtures used for calculation of yields are collected in Supporting Information File 1): good yields were observed for 4-methylacetophenone (**2b**, 97%, Figure S9), 3-methoxyacetophenone (**2c**, 87%, Figure S10), 4-chloroacetophenone (**2d**, 76%, Figure S11), and 4-(trifluoromethyl)acetophenone (**2e**, 86%, Figure S12) and a moderate yield was obtained for 2-acetonaphthone (**2j**, 50%, Figure S13) demonstrating the general applicability of ***m*-TPEWP5**
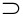
**G-EsY** as an efficient photocatalyst.

**Figure 5:**
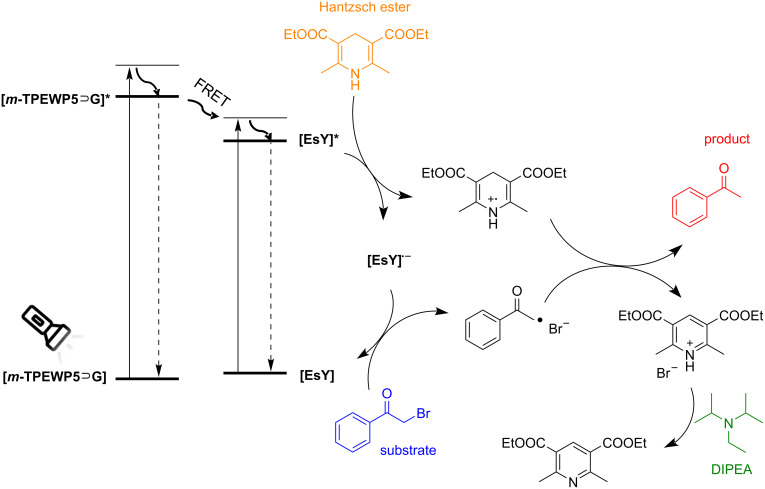
Proposed mechanism for the 2-bromo-1-phenylethanone dehalogenation reaction mediated by ***m*-TPEWP5**
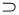
**G-EsY** nanoassembly as a photocatalyst.

To understand the process for this photocatalytic dehalogenation reaction, a possible reaction mechanism is proposed in Figure 5 [37]. Upon light irradiation, the ground state of **m-TPEWP5**
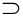
**G** donor absorbs light energy and changes to the excited state (TPEWP5
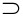
G*) energy level. Through energy transfer from TPEWP5
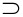
G* to ground state **EsY** the latter undergoes excitation to the excited state EsY* and is reduced by the Hantzsch ester to generate the radical anion **EsY^•−^**. Subsequently, electron transfer from **EsY^•−^** to the substrate α-bromoacetophenone (**1a**) gives the corresponding acetophenone radical, whilst **EsY^•−^** is oxidized to **EsY**. The acetophenone radical combines with a H-atom abstracted from the radical cation of the Hantzsch ester to form acetophenone (**2a**) as the final product and diethyl 2,6-dimethylpyridine-3,5-dicarboxylate after deprotonation in the presence of the base DIPEA.

## Conclusion

In conclusion, we have fabricated a simple and efficient supramolecular photocatalytic system based on host–guest self-assembled ***m*-TPEWP5**
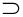
**G** donor and **EsY** as acceptor. Briefly, the ***m*-TPEWP5** host and **G** guest molecule were involved in the inclusion complex and further self-assembled to form worm-like supramolecular nanostructures, which displayed an AIE effect via restricted phenyl-ring rotation of ***m*-TPEWP5**. After that, the negative **EsY** acceptor was loaded on the positively charged surface of the ***m*-TPEWP5**
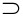
**G** donor assembly to form a nanorod structure, which exhibited moderate FRET efficiency at a 2:1 molar ratio of the donor/acceptor. Inspired by photosynthesis and followed by the energy transfer process, the ***m*-TPEWP5**
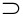
**G-EsY** supramolecular nanorod assembly could be employed as a nanoreactor for a photocatalytic dehalogenation reaction, i.e., debromination of 2-bromo-1-phenylethanone derivatives with high yields and short reaction time in an aqueous solution. Based on the above results, the fabricated AIE-emissive FRET system with chiral guest can be further utilized for asymmetric catalysis in water, which is currently underway in our laboratory.

## Supporting Information

File 1 Experimental details, NMR spectra, host–guest interaction, FRET, and other materials.

## References

[1] McDermott G, Prince S M, Freer A A, Hawthornthwaite-Lawless A M, Papiz M Z, Cogdell R J, Isaacs N W (1995). Nature.

[2] Holt N E, Zigmantas D, Valkunas L, Li X-P, Niyogi K K, Fleming G R (2005). Science.

[3] Polívka T, Frank H A (2010). Acc Chem Res.

[4] Scholes G D, Fleming G R, Olaya-Castro A, van Grondelle R (2011). Nat Chem.

[5] Xiao T, Zhong W, Zhou L, Xu L, Sun X-Q, Elmes R B P, Hu X-Y, Wang L (2019). Chin Chem Lett.

[6] Wang K, Velmurugan K, Li B, Hu X-Y (2021). Chem Commun.

[7] Barber J (2009). Chem Soc Rev.

[8] Teunissen A J P, Pérez-Medina C, Meijerink A, Mulder W J M (2018). Chem Soc Rev.

[9] Jia P-P, Xu L, Hu Y-X, Li W-J, Wang X-Q, Ling Q-H, Shi X, Yin G-Q, Li X, Sun H (2021). J Am Chem Soc.

[10] Wang K, Jordan J H, Velmurugan K, Tian X, Zuo M, Hu X-Y, Wang L (2021). Angew Chem, Int Ed.

[11] Zuo M, Velmurugan K, Wang K, Tian X, Hu X-Y (2021). Beilstein J Org Chem.

[12] Pochan D, Scherman O (2021). Chem Rev.

[13] Hartgerink J D, Beniash E, Stupp S I (2001). Science.

[14] Huang J, Yu Y, Wang L, Wang X, Gu Z, Zhang S (2017). ACS Appl Mater Interfaces.

[15] Guo S, Song Y, He Y, Hu X-Y, Wang L (2018). Angew Chem, Int Ed.

[16] Huo M, Ye Q, Che H, Wang X, Wei Y, Yuan J (2017). Macromolecules.

[17] Peng H-Q, Chen Y-Z, Zhao Y, Yang Q-Z, Wu L-Z, Tung C-H, Zhang L-P, Tong Q-X (2012). Angew Chem, Int Ed.

[18] Chadha G, Yang Q-Z, Zhao Y (2015). Chem Commun.

[19] Pallavi P, Sk B, Ahir P, Patra A (2018). Chem – Eur J.

[20] Chen P-Z, Weng Y-X, Niu L-Y, Chen Y-Z, Wu L-Z, Tung C-H, Yang Q-Z (2016). Angew Chem, Int Ed.

[21] Ling Q, Cheng T, Tan S, Huang J, Xu L (2020). Chin Chem Lett.

[22] Li Y, Rajasree S S, Lee G Y, Yu J, Tang J-H, Ni R, Li G, Houk K N, Deria P, Stang P J (2021). J Am Chem Soc.

[23] Kim H-J, Nandajan P C, Gierschner J, Park S Y (2018). Adv Funct Mater.

[24] Zhang D, Liu Y, Fan Y, Yu C, Zheng Y, Jin H, Fu L, Zhou Y, Yan D (2016). Adv Funct Mater.

[25] Sun G, Qian W, Jiao J, Han T, Shi Y, Hu X-Y, Wang L (2020). J Mater Chem A.

[26] Petroselli M, Chen Y-Q, Rebek J, Yu Y (2021). Green Synth Catal.

[27] Ogoshi T, Yamafuji D, Yamagishi T-a, Brouwer A M (2013). Chem Commun.

[28] Wang X-H, Song N, Hou W, Wang C-Y, Wang Y, Tang J, Yang Y-W (2019). Adv Mater (Weinheim, Ger).

[29] Zhang D, Yu W, Li S, Xia Y, Li X, Li Y, Yi T (2021). J Am Chem Soc.

[30] Hao M, Sun G, Zuo M, Xu Z, Chen Y, Hu X-Y, Wang L (2020). Angew Chem, Int Ed.

[31] Tian X, Zuo M, Niu P, Velmurugan K, Wang K, Zhao Y, Wang L, Hu X-Y (2021). ACS Appl Mater Interfaces.

[32] Velmurugan K, Murtaza A, Saeed A, Li J, Wang K, Zuo M, Liu Q, Hu X-Y (2022). CCS Chem.

[33] Luo J, Xie Z, Lam J W Y, Cheng L, Chen H, Qiu C, Kwok H S, Zhan X, Liu Y, Zhu D (2001). Chem Commun.

[34] Le Guével X, Hötzer B, Jung G, Schneider M (2011). J Mater Chem.

[35] Velmurugan K, Thamilselvan A, Antony R, Kannan V R, Tang L, Nandhakumar R (2017). J Photochem Photobiol, A.

[36] Prabakaran G, Velmurugan K, Vickram R, David C I, Thamilselvan A, Prabhu J, Nandhakumar R (2021). Spectrochim Acta, Part A.

[37] Sun G, Zuo M, Qian W, Jiao J, Hu X-Y, Wang L (2021). Green Synth Catal.

