# Supplementary material for: Tetraphenylethylene-embedded pillar[5]arene-based orthogonal self-assembly for efficient photocatalysis in water
Source: Beilstein J Org Chem. 2022 Apr 13;18:429–37. doi: 10.3762/bjoc.18.45 (PMC9039527; doi:10.3762/bjoc.18.45)
Supplement: File 1 — Experimental details, NMR spectra, host–guest interaction, FRET, and other materials. [file Beilstein_J_Org_Chem-18-429-s001.pdf]

BEILSTEIN JOURNAL OF ORGANIC CHEMISTRY

Supporting Information

for

Tetraphenylethylene-embedded pillar[5]arene-based orthogonal self-assembly for efficient photocatalysis in water

Zhihang Bai, Krishnasamy Velmurugan, Xueqi Tian, Minzan Zuo, Kaiya Wang and Xiao-Yu Hu

Beilstein J. Org. Chem. **2022,** *18,* 429–437. doi:10.3762/bjoc.18.45

Experimental details, NMR spectra, host–guest interaction, FRET, and other materials

License and Terms: This is a supporting information file under the terms of the Creative Commons Attribution License (https://creativecommons.org/licenses/by/4.0). Please note that the reuse, redistribution and reproduction in particular requires that the author(s) and source are credited and that individual graphics may be subject to special legal provisions.
The license is subject to the *Beilstein Journal of Organic Chemistry* terms and conditions: (https://www.beilstein-journals.org/bjoc/terms)

Table of contents

1. General information and experimental procedure..S2

2. Host-guest interaction between **m-TPEWP5** and **G** S4

3. Investigation of the FRET efficiency in **m-TPEWP5** ⊃ **G** assembly S6

4. Photocatalytic debromination reactions based on **m-TPEWP5** ⊃ **G+EsY** assembly S7

5. References...S13

1. General information and experimental procedure

All common available reagents and solvents were purchased from Energy Chemical Reagent Co., Ltd. and used without further purification. Column chromatography was performed with silica gel (200–300 mesh) produced by Shanghai Titan Scientific Co., Ltd. Yields were given as isolated or calculated yields. NMR spectra were recorded with a Bruker 400 MHz spectrometer. UV–vis absorption and fluorescence spectra were taken on a SHIMADZU UV-1700 UV and Angdong F-380 Spectrometer, respectively. Transmission electron microscope (TEM) analysis was performed on a JEM-2100 instrument.

Experimental procedure: The detailed synthetic routes of the host molecule ***m*-TPEWP5** and guest molecule **G** are shown in Scheme S1 and Scheme S2.

Synthesis of *m*-TPEWP5.

***m*-TPEWP5** was synthesized according to our previously reported procedures (Scheme S1). The characterization data can be found in our previous work.[S1]

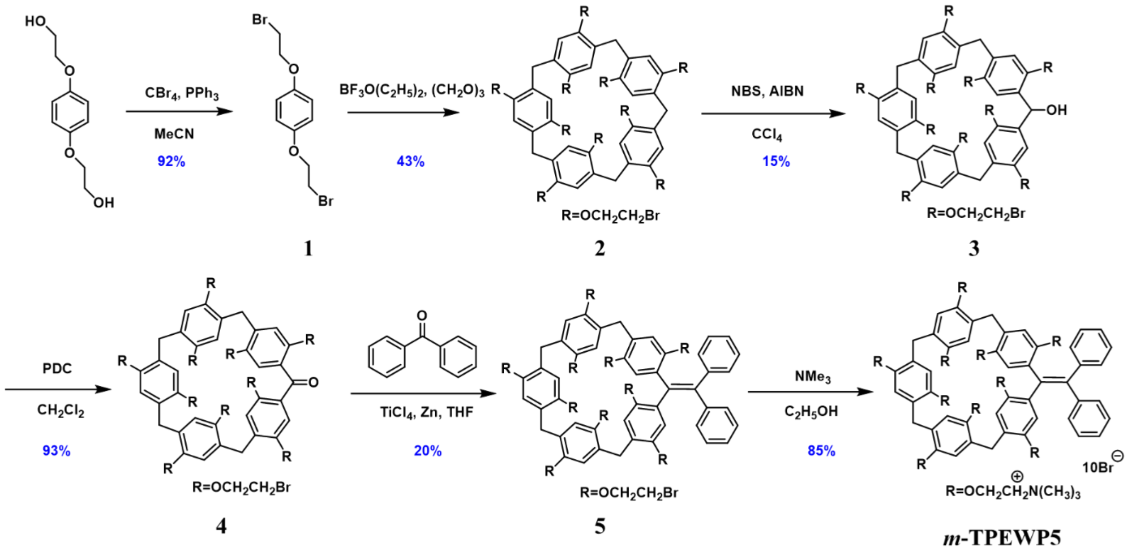

Scheme S1: Synthetic route of ***m*-TPEWP5**

Synthesis of compound G[S2]

A mixture of benzoyl-L-alanine (100 mg, 0.00241 mmol) and excess $NH_3 \cdot H_2O$ (25.0 mL, 7.0 mol) was stirred at room temperature for 6 h. The solvent was evaporated under reduced pressure and the residue was dried in vacuum. The product was obtained as a white solid (102 mg, 100%).^{1}H NMR (400 MHz, D_2O, 298 K): δ (ppm): 7.74 (d, 2H), 7.57-7.53 (t, 1H), 7.48-7.45 (t, 1H), 4.29 (d, 1H), 1.39 (d, 3H).

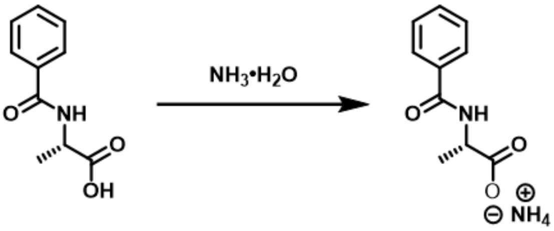

Scheme S2: Synthetic route of **G**

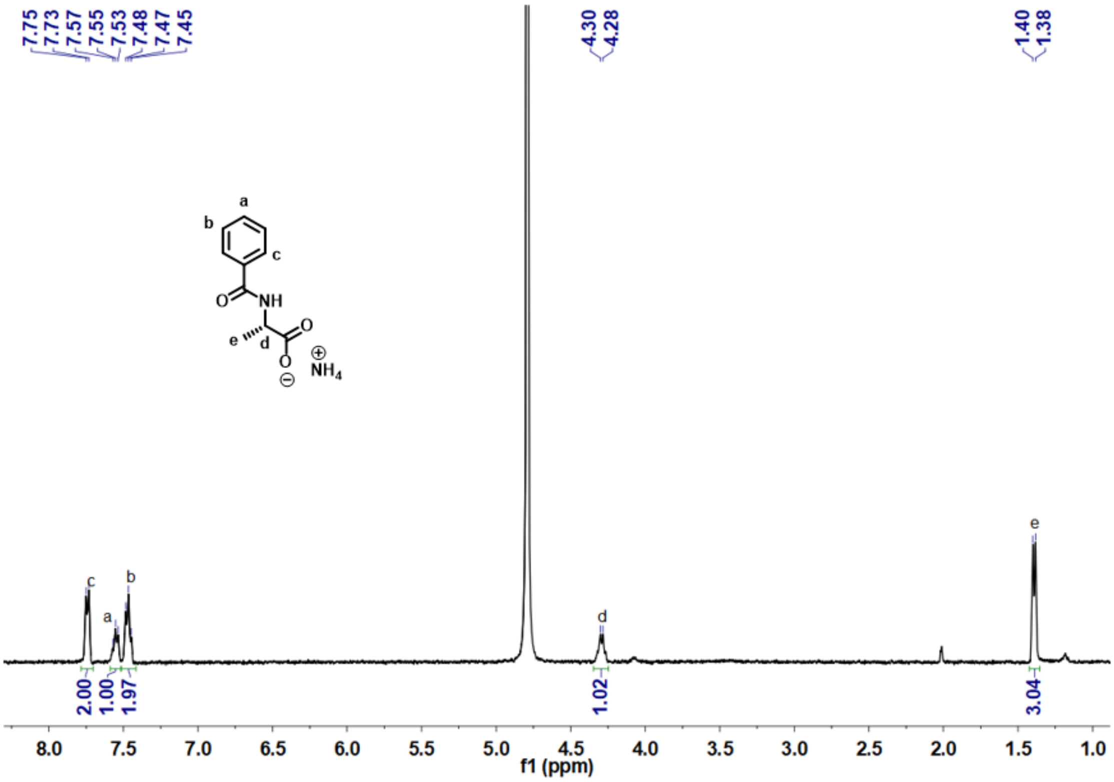

Figure S1: ^{1}H NMR spectrum (400 MHz, D_2O, 298 K) of compound **G**.

2. Host–guest interaction between *m*-TPEWP5 and G

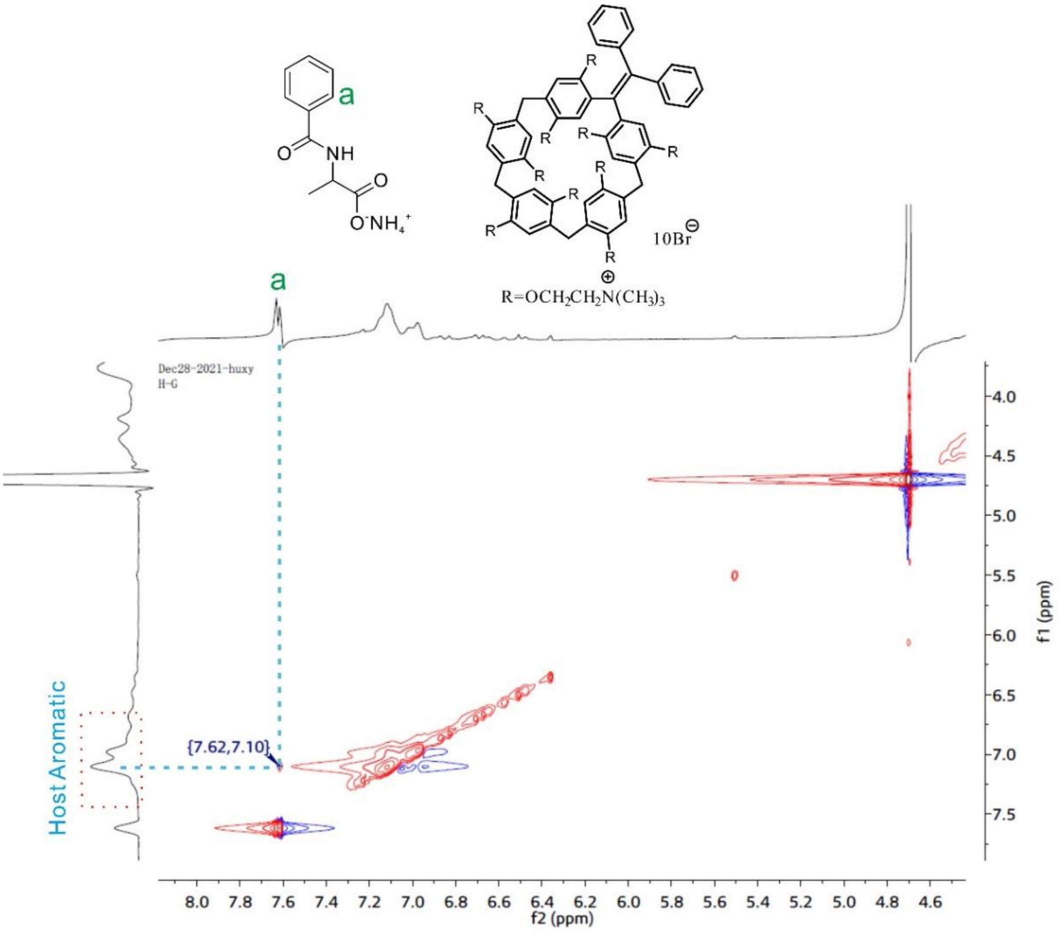

Figure S2: 2D NOESY NMR (400 MHz, D_2O, 298 K) spectrum of the ***m*-TPEWP5**$\supset$ **G** complex (***m*-TPEWP5** (1.0 mM) + **G** (1.0 mM)).

Job’s plot for *m*-TPEWP5 ⊃ G

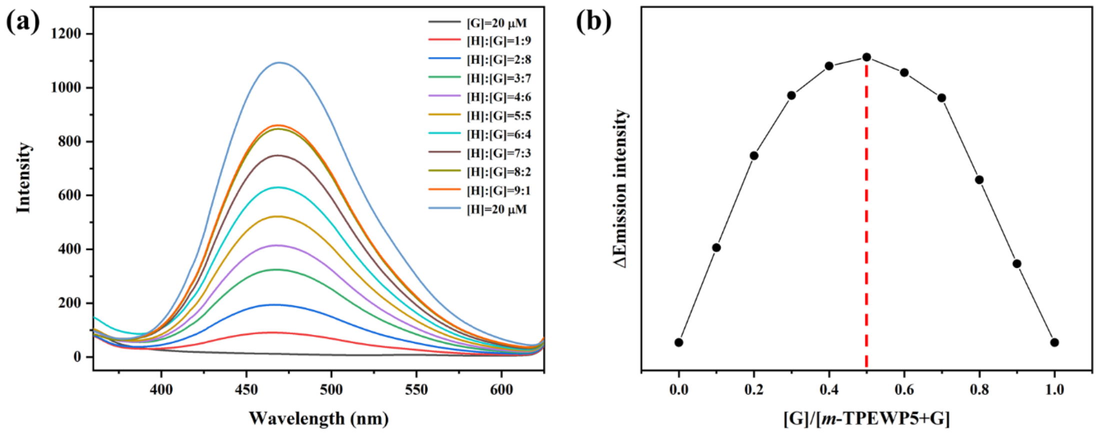

Figure S3: (a) Fluorescence spectra of ***m*-TPEWP5 ⊃ G** at different molar ratios in water with a total concentration of **[*m*-TPEWP5]** + **[G]** = 2 × 10^{-5} M; (b) Job’s plot of ***m*-TPEWP5 ⊃ G** complex showing a 1:1 stoichiometry between ***m*-TPEWP5** and **G** by plotting the emission differences at 470 nm (a characteristic emission peak of ***m*-TPEWP5**) against the mole fraction of **G**; the excitation wavelength was 320 nm.

Determination of the association constant (K_a) for *m*-TPEWP5 ⊃ G

To determine the association constant between ***m*-TPEWP5** and **G**, UV–vis measurements were carried out in solutions, which had a constant concentration of ***m*-TPEWP5** (0.1 mM) and varying concentrations of **G**. By a linear curve-fitting method, the association constant between the **G** and ***m*-TPEWP5** was calculated. The linear curve-fitting was based on the following equation[S3]

Benesi–Hildebrand equation for the 1:1 stoichiometric ratio

$$\frac{1}{A-A_0}=\frac{1}{A'-A_0}+\frac{1}{K_a\,[A-A_0][G]} \qquad (1)$$

Here, A_0, A, and A′ are represented as the absence, presence and saturated absorption of guest (**G**) with host (***m*-TPEWP5**), and [**G**] is the concentration of the guest. Plotting of $1/A-A_0$ versus 1/[**G**] showed a linear relationship, which also strongly supported the 1:1 stoichiometry of ***m*-TPEWP5 ⊃ G** complex, and the association constant (K_a) was calculated to be 8.62 × 10^4 M^{-1}.

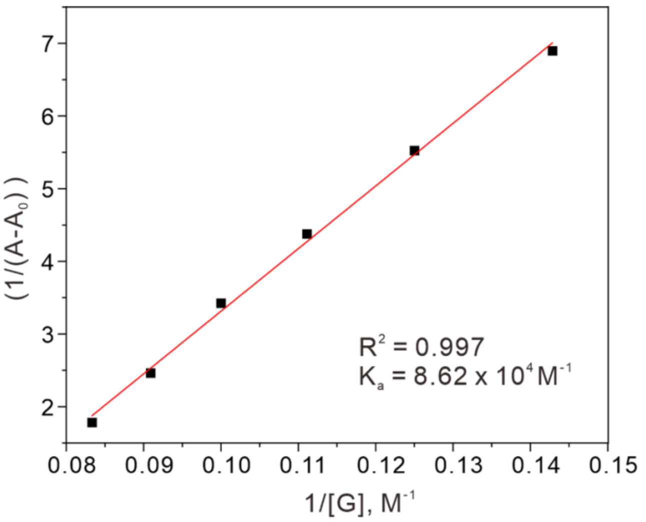

Figure S4: The UV absorption changes of ***m*-TPEWP5** upon the addition of **G**. The red solid line was obtained from the non-linear curve-fitting using Eq.1. The association constant (K_a) of ***m*-TPEWP5** and **G** was calculated to be 8.62 × 10^4 M^{-1}.

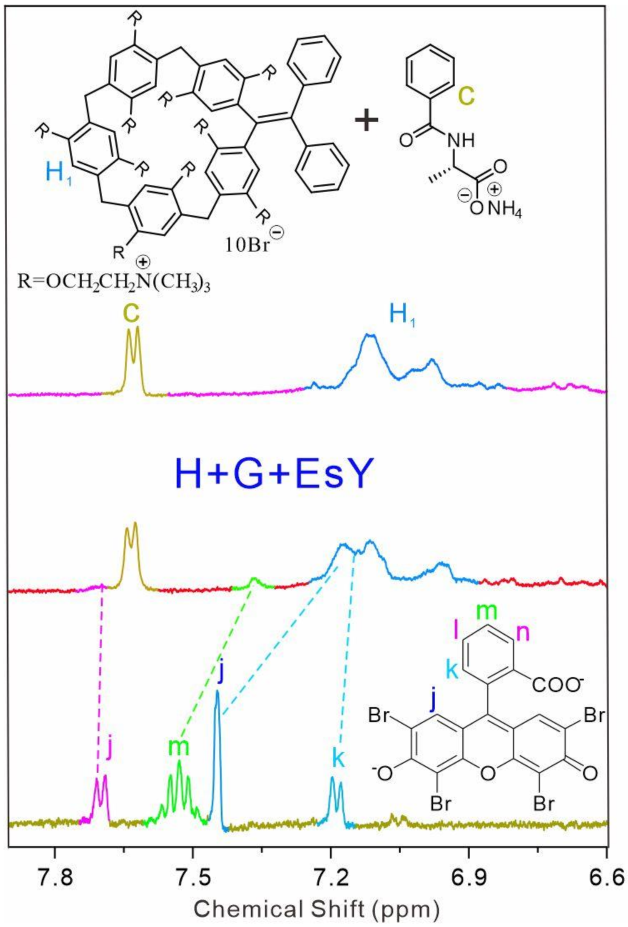

Figure S5: ^{1}H NMR (400 MHz, D_2O, 298 K) spectra: ***m*-TPEWP5** (1.0 mM) + **G** (1.0 mM), ***m*-TPEWP5** (1.0 mM) + **G** (1.0 mM) + **EsY** (1.0 mM), and **EsY** (1.0 mM).

3. Investigation of the FRET efficiency in *m*-TPEWP5⊃G assembly

Energy-transfer efficiency calculation

The energy-transfer efficiency (Φ_{ET}) was calculated from excitation fluorescence spectra through the equation,

$\Phi_{ET} = 1 - I_{DA} / I_D$

Where I_{DA} and I_D are the fluorescence intensities of the ***m*-TPEWP5⊃G-EsY** assembly (donor and acceptor) and ***m*-TPEWP5⊃G** assembly (donor), respectively, when excited at 320 nm. The energy-transfer efficiency (Φ_{ET}) was calculated to be 31% in aqueous solution, measured under the condition of [***m*-TPEWP5**] = 1 × 10^{-5} M, [**G**] = 1 × 10^{-5} M, [**EsY**] = 1 × 10^{-5} M.

4. Photocatalytic debromination reactions based on *m*-TPEWP5⊃G+EsY assembly

Method 1:

Since the formed nanorod assembly is water-soluble, hence, $CDCl_3$ was selected to extract the product and substrate from the reaction solution after the completion of reaction. In this way, it contains the characteristic peaks of Hantzsch ester and DIPEA, but we only selected the -CH_3 of the product and the -CH_2 of the substrate for calibration. And relative measurements were used to calculate yields: $\eta = \frac{\frac{A1}{n1}}{\frac{A1}{n1}+\frac{A2}{n2}}\%$

Here, A_1 is the integral area of the product -CH_3, n_1 is the number of protons in the characteristic peak of the product, A_2 is the integral area of the substrate -CH_2, n_2 is the number of protons in the characteristic peak of the substrate.

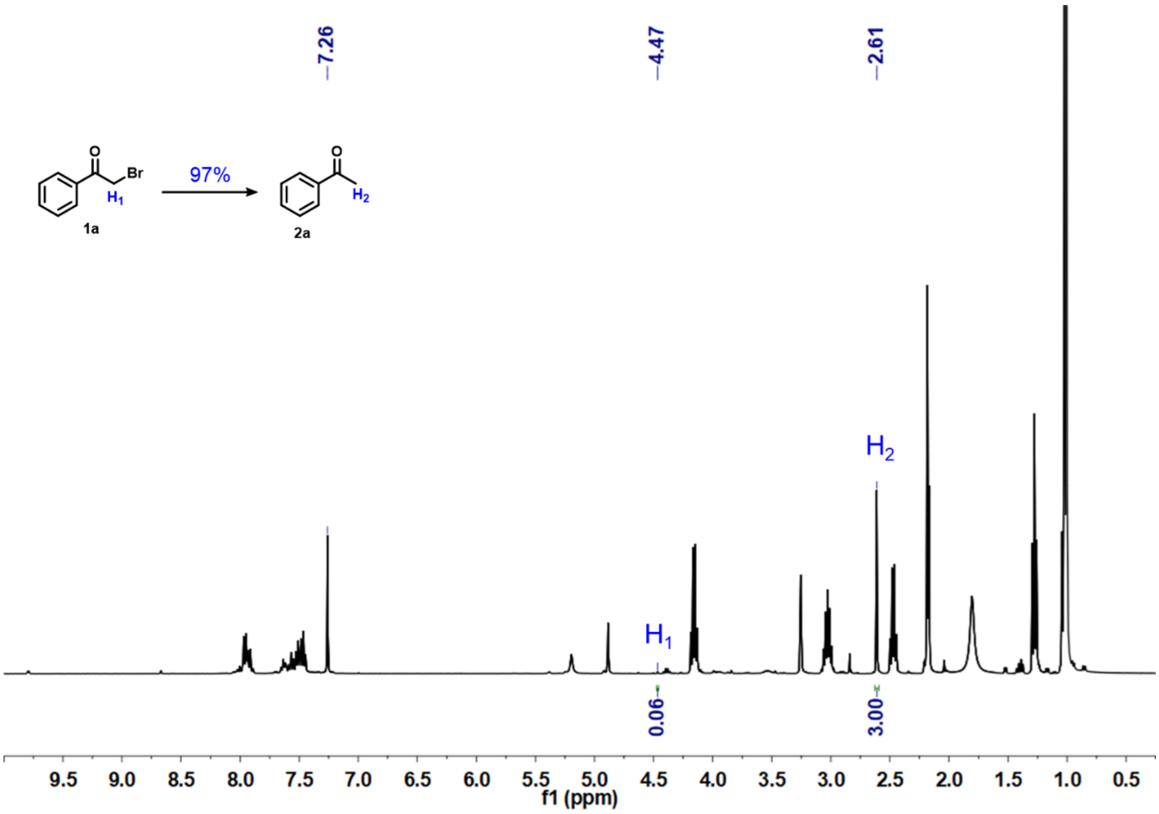

Figure S6: ^{1}H NMR (400 MHz, $CDCl_3$, 298 K) of reaction mixture containing **1a** and **2a** in the presence of ***m*-TPEWP5⊃G-EsY** nanocomposite.

Method 2:

Since the formed nanorod assembly is water-soluble, hence, $CDCl_3$ was selected to extract the product and substrate from the reaction solution after the completion of reaction. The signal peaks of 1,3,5-trimethoxybenzene in ^{1}H NMR are strong and do not overlap with the product peaks. Therefore, this compound was selected as an internal standard and added into the reaction mixture. We only selected the -CH_3 of the product and the -OCH_3 of the internal standard for calibration. The ratio between product and internal standard: $r_{A/Is}=\frac{\frac{B1}{m1}}{\frac{B2}{m2}}$, $n_{IS}=\frac{mIS}{MIS}$ can be obtained by weighing a certain amount of internal standard. Then, the following equation was used to calculate the exact data.

$$n_A = n_{IS} \cdot r_{A/IS}$$

Finally, the yield of the product was obtained by using the following equation: $\eta=\frac{nA}{nth}$ %

Here, B_1 is the integral area of the product -CH_3, m_1 is the number of protons in the

characteristic peak of the product, B_2 is the integral area of the internal standard -OCH_3, m_2 is the number of protons in the characteristic peak of the internal standard. m_{IS} is the weight of internal standard, M_{IS} is the relative molecular mass of internal standard, n_{th} is theoretical yield of product **2a** (0.1 mmol).

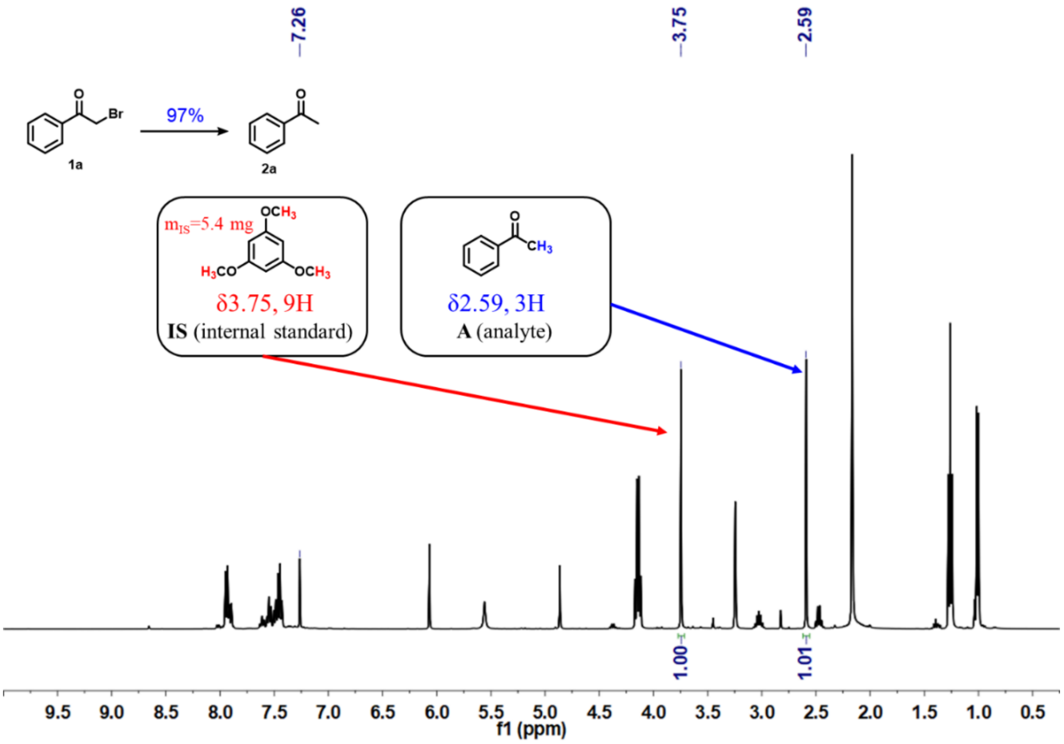

Figure S7: ^1H NMR (400 MHz, $CDCl_3$, 298 K) of the reaction mixture containing 1,3,5-trimethoxybenzene and **2a** in the presence of ***m*-TPEWP5⊃G-EsY** nanocomposite.

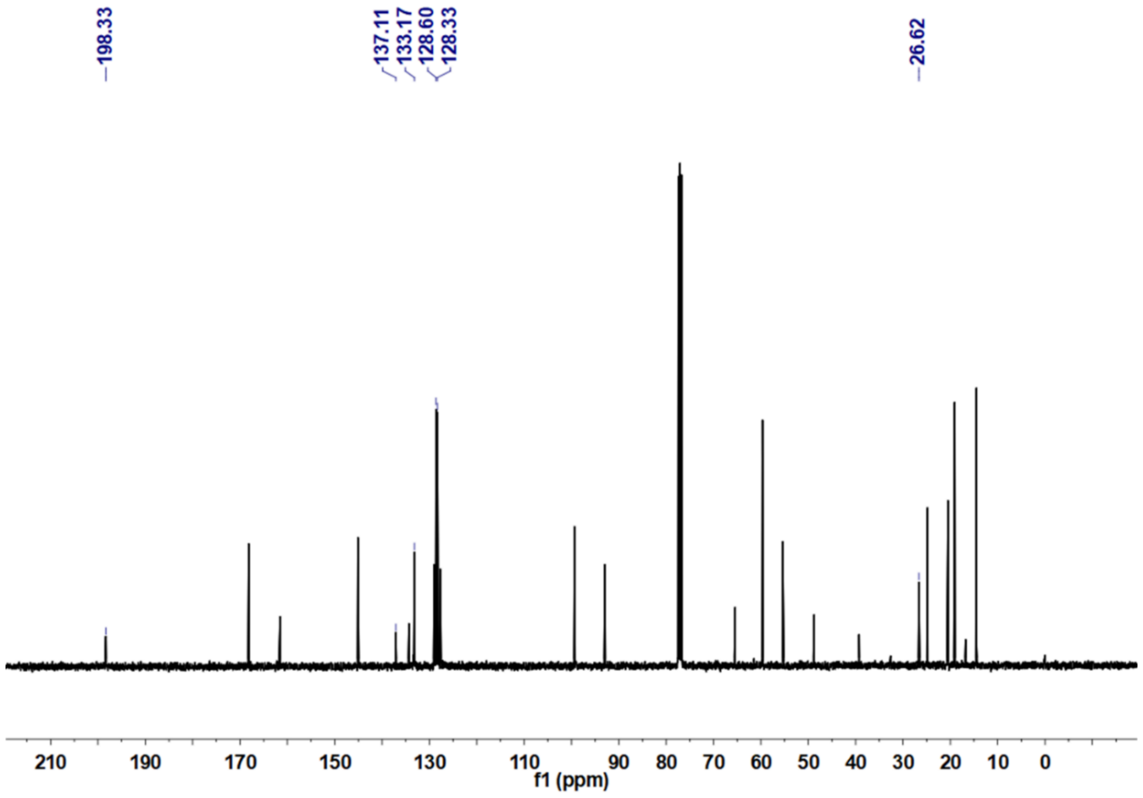

Figure S8: ^{13}C NMR (400 MHz, $CDCl_3$, 298 K) of reaction mixture containing **1a** and **2a** in the presence of ***m*-TPEWP5 ⊃ G-EsY** nanocomposite.

The following calculations for products **2b**, **2c**, **2d**, **2e**, and **2f** were performed by **Method 1**:

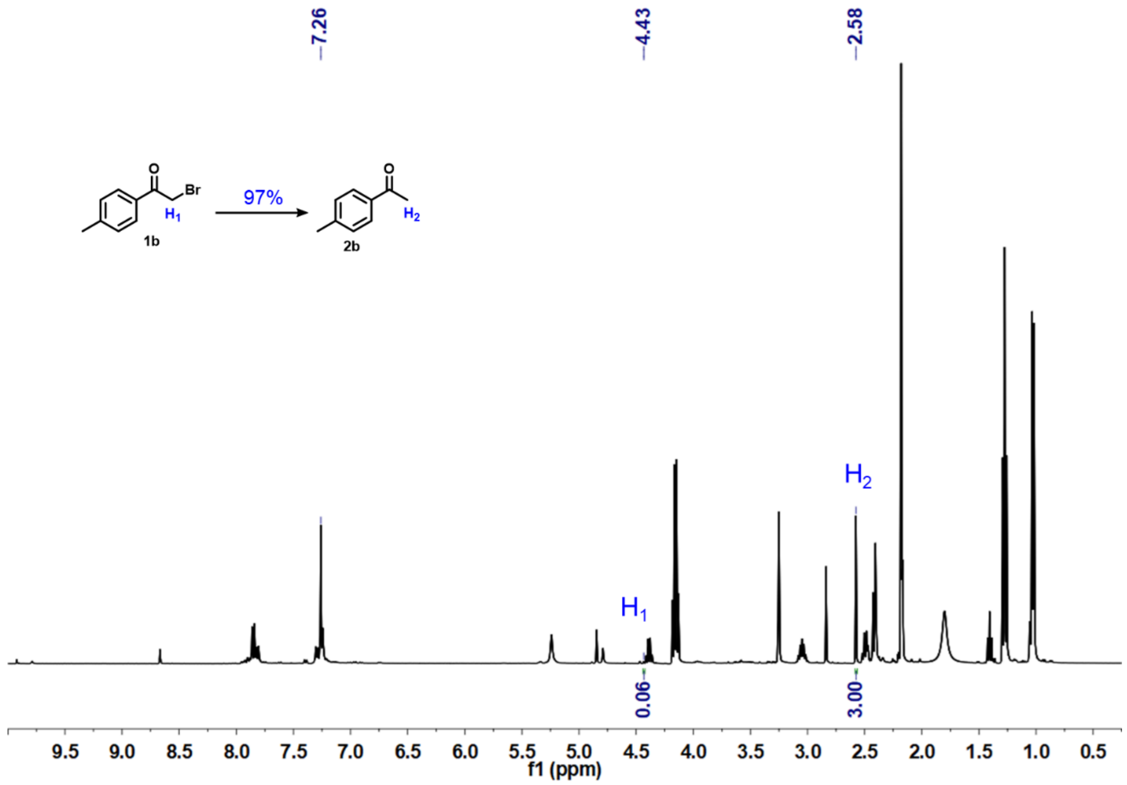

Figure S9: ^{1}H NMR (400 MHz, $CDCl_3$, 298 K) of the reaction mixture containing **1b** and **2b** in the presence of ***m*-TPEWP5** ⊃ **G-EsY** nanocomposite.

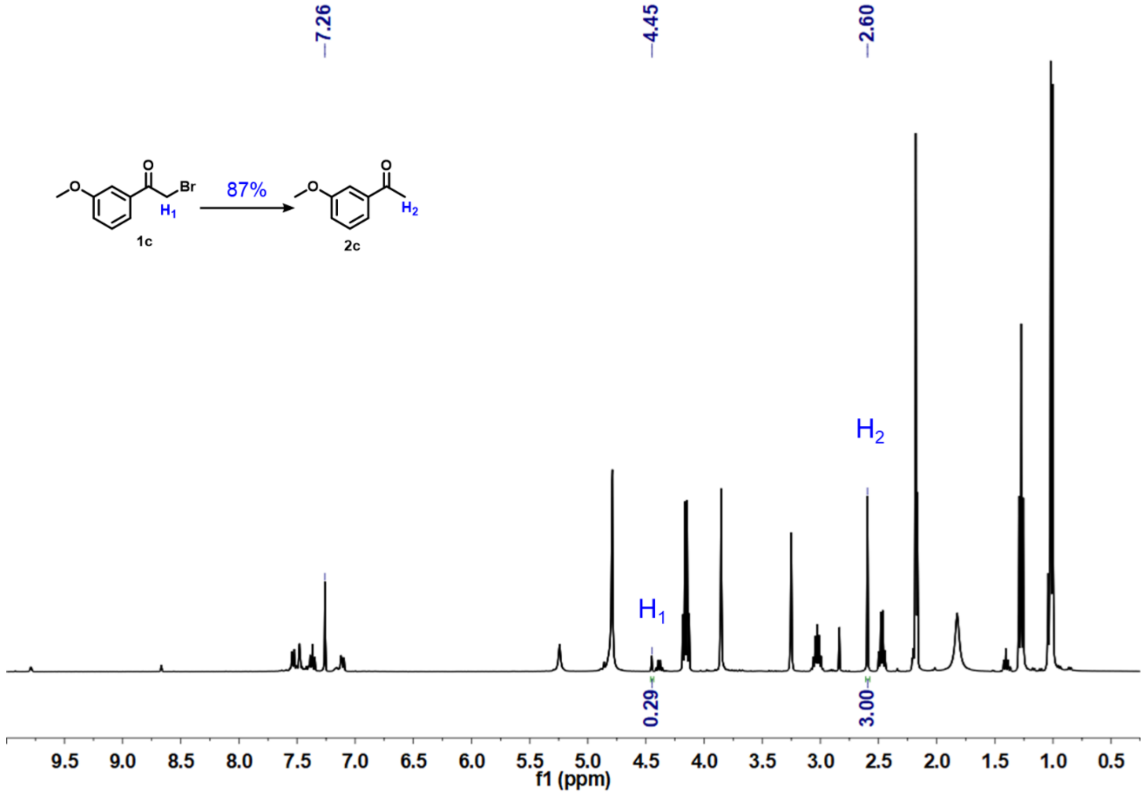

Figure S10: ^{1}H NMR (400 MHz, $CDCl_3$, 298 K) of the reaction mixture containing **1c** and **2c** in the presence of ***m*-TPEWP5** ⊃ **G-EsY** nanocomposite.

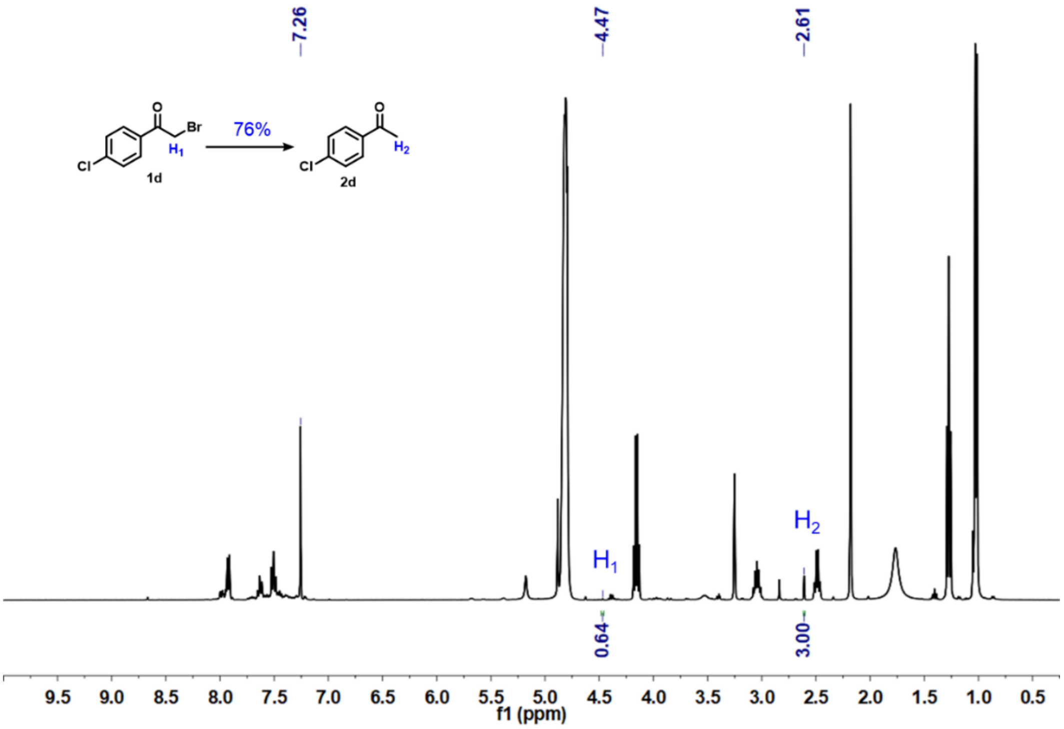

Figure S11: ^{1}H NMR (400 MHz, $CDCl_3$, 298 K) of the reaction mixture containing **1d** and **2d** in the presence of ***m*-TPEWP5** ⊃ **G-EsY** nanocomposite.

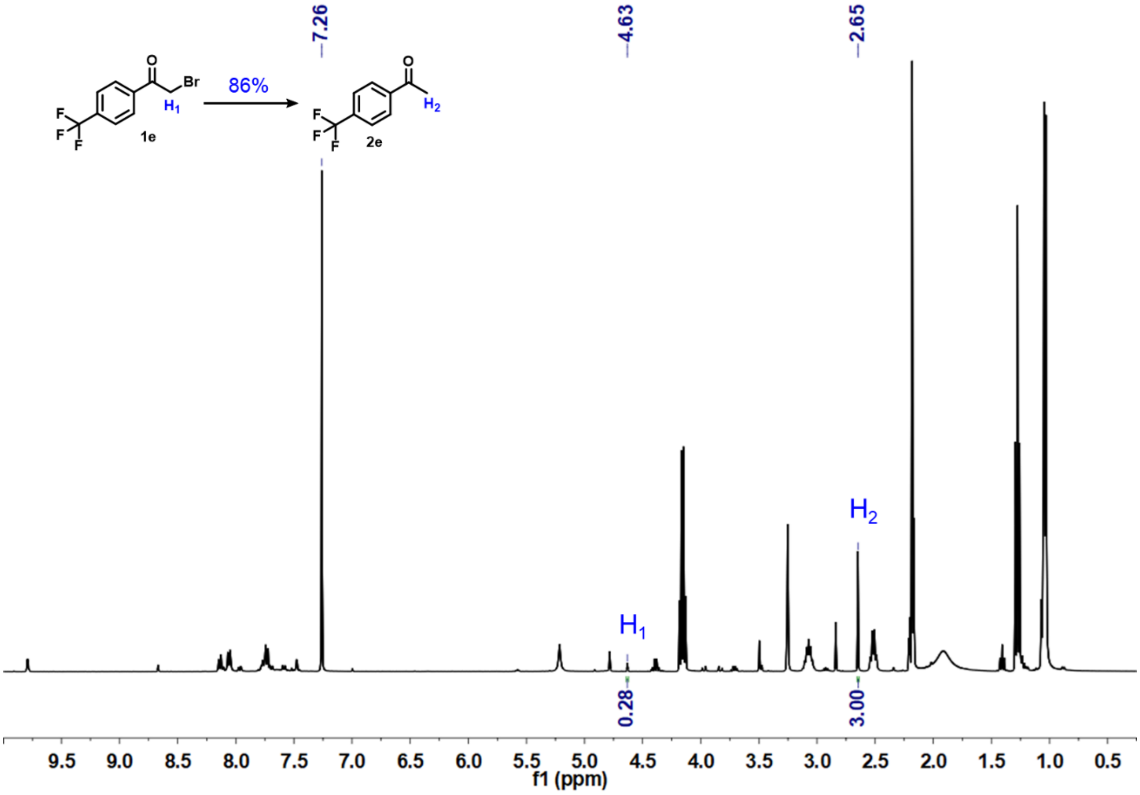

Figure S12: ^{1}H NMR (400 MHz, $CDCl_3$, 298 K) of the reaction mixture containing **1e** and **2e** in the presence of ***m*-TPEWP5** ⊃ **G-EsY** nanocomposite.

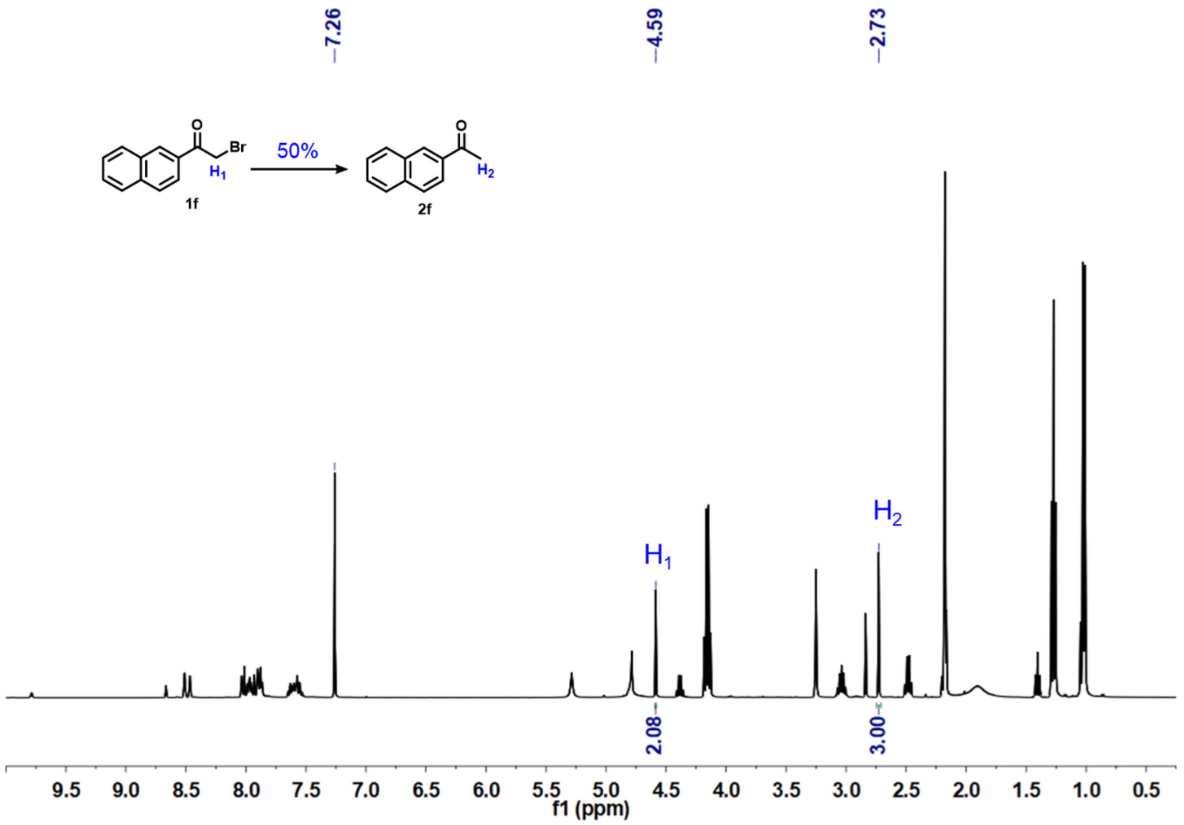

Figure S13: ^{1}H NMR (400 MHz, $CDCl_3$, 298 K) of the reaction mixture containing **1f** and **2f** in the presence of ***m*-TPEWP5 ⊃ G-EsY** nanocomposite.

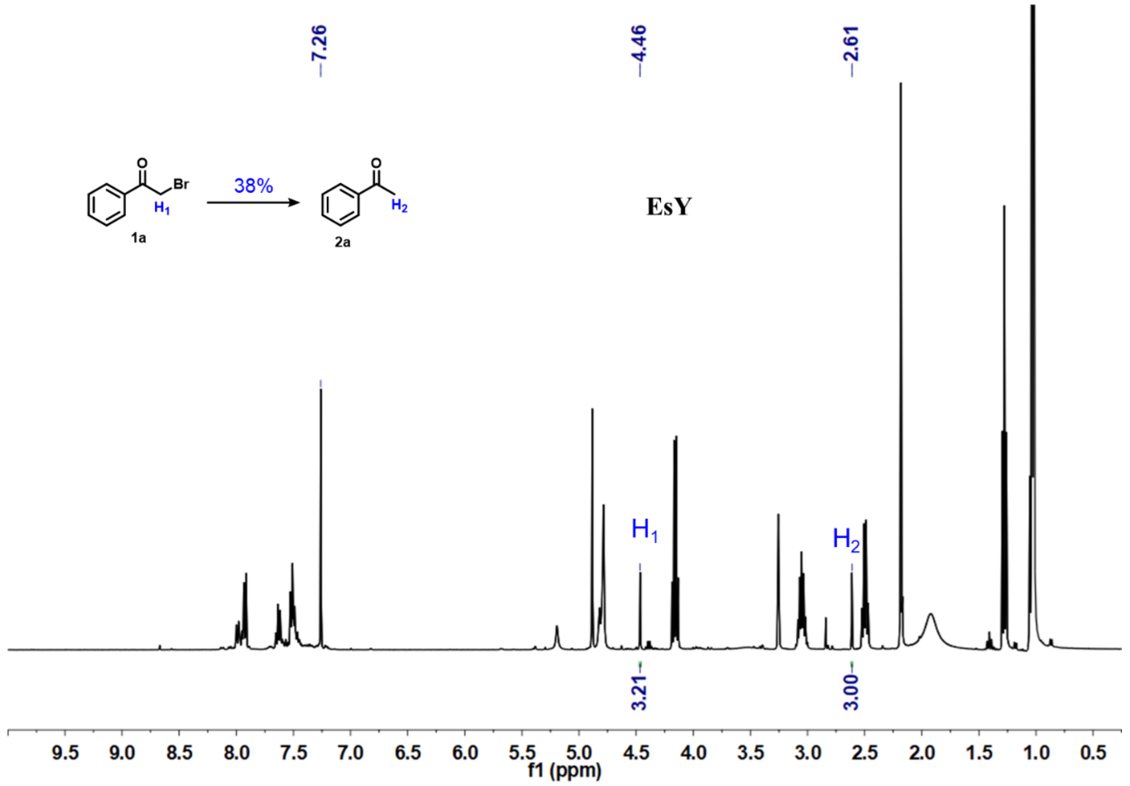

Figure S14: ^{1}H NMR (400 MHz, $CDCl_3$, 298 K) of the reaction mixture containing **1a** and **2a** in the presence of **EsY** only.

5. References

[S1]Tian, X.; Zuo, M.; Niu, P.; Velmurugan, K.; Wang, K.; Zhao, Y.; Wang, L.; Hu, X.-Y. *ACS Appl. Mater. Interfaces*, **2021**, *13*, 37466-37474.

[S2] Velmurugan, K.; Murtaza, A.; Saeed, A.; Li, J.; Wang, K.; Zuo, M.; Liu, Q.; Hu, X.-Y. *CCS Chem.*, **2022**, in press. DOI: 10.31635/ccschem.022.202101749

[S3] Velmurugan, K.; Prabhu, J.; Tang, L.; Chidambaram, T.; Noel, M.; Radhakrishnan, S.; Nandhakumar, R. *Anal. Methods*, **2014**, *6*, 2883-2888.
